# DEFENS - Drug Exposure Feedback and Education for Nurses’ Safety: study protocol for a randomized controlled trial

**DOI:** 10.1186/s13063-015-0674-5

**Published:** 2015-04-17

**Authors:** Christopher R Friese, Kari Mendelsohn-Victor, Bo Wen, Duxin Sun, Kathleen Sutcliffe, James J Yang, David L Ronis, Marjorie C McCullagh, For the DEFENS Study Investigators

**Affiliations:** University of Michigan School of Nursing, 400 North Ingalls, #4162, Ann Arbor, MI USA; University of Michigan College of Pharmacy, Pharmacokinetics Core, Ann Arbor, MI USA; Johns Hopkins University Carey School of Business and School of Medicine, Baltimore, MD USA

**Keywords:** Oncologic nursing, Antineoplastic drugs, Cluster randomized trials, Occupational safety and health, Web-based interventions

## Abstract

**Background:**

Three decades of research findings have documented the health effects of handling hazardous drugs. Oncology nurses are vulnerable due to frequent administration of antineoplastics, low adherence to equipment use, reported barriers to use, and perceived low risk of health effects. No interventions have been tested in a controlled, multi-site trial to increase nurses’ use of protective equipment when handling hazardous drugs. The Drug Exposure Feedback and Education for Nurses’ Safety (DEFENS) study will compare the efficacy of education (control) versus an audit and feedback intervention (treatment) on nurses’ self-reported use of personal protective equipment when handling hazardous drugs. The treatment intervention will include tailored messages based on nurses’ reported barriers to protective equipment use.

**Methods/Design:**

The DEFENS Study is a cluster randomized controlled trial. We are enrolling cancer centers and will recruit nurse participants in April 2015. Eligible cancer centers employ at least 20 eligible registered nurses in the chemotherapy infusion setting and have on-site phlebotomy resources. Eligible participants are nurses who work at least 0.40 full-time equivalent hours in the chemotherapy infusion setting and have not received an antineoplastic drug for a health problem in the past year. An encrypted, user-authenticated website will administer surveys and deliver control and treatment interventions. The primary endpoint is the change in score on nurses’ reports of the Revised Hazardous Drug Handling Questionnaire between baseline and approximately 18 months later. A baseline survey is completed after informed consent and is repeated 18 months later. Nurses in all sites who experience a drug spill will also report incidents as they occur; these reports inform the treatment intervention. Plasma will be obtained at baseline, approximately 18 months later (the primary endpoint), and with drug spill occurrences to measure hazardous drugs levels and to inform the treatment intervention. Potential mediators include knowledge of hazardous drug handling and perceived risk of drug exposure. We will examine whether personal factors and organizational factors moderate the intervention effects.

**Trial registration:**

Clinicaltrials.gov NCT02283164, registered 31 October 2014.

**Electronic supplementary material:**

The online version of this article (doi:10.1186/s13063-015-0674-5) contains supplementary material, which is available to authorized users.

## Background

For over three decades, scientists have documented the pernicious effects of handling hazardous drugs such as antineoplastics [1-5]. Reports have identified the following health effects: acute nausea and vomiting, reproductive difficulties, cancer, and myelodysplastic syndrome. In 2004, the National Institute for Occupational Safety and Health (NIOSH) issued an alert that summarized the evidence and recommended that health care settings and employees adopt practices to minimize the risk of handling potentially hazardous drugs [6]. The Oncology Nursing Society [7] and the American Society of Health-System Pharmacists [8] published guidelines on hazardous drug handling. The NIOSH 2004 recommendations are now included in the 2013 American Society of Clinical Oncology/Oncology Nursing Society chemotherapy administration safety standards [9].

Published guidelines include the use of personal protective equipment (PPE), comprised of two pairs of chemotherapy-tested gloves, single-use disposable gowns, eye protection during specific activities, and respiratory protection when vapor exposure is possible. However, adoption of these guidelines is suboptimal in clinical settings [10]. A statewide survey revealed that 16.9% of ambulatory oncology nurses reported skin or eye exposure to chemotherapy in the past year [11]. Increased exposure is associated with higher nursing workloads and poorer nurse practice environments. Oncology nurses deliver an astounding volume of chemotherapy; US estimates suggest over 18 million doses of chemotherapy are administered annually in the United States, primarily by nurses [12].

Audit and feedback is an established intervention to support clinician practice change. Successful audit and feedback interventions include education and periodic reminders [13]. Systematic reviews have identified improvements in clinician practice after feedback interventions [14]. Using a pre-post design, one Malaysian study reported increased scores on safe handling knowledge, beliefs, and observed practices for 96 inpatient nurses who completed an educational module on hazardous drug handling [15]. The absence of multi-site, controlled intervention studies to improve PPE use in ambulatory oncology nurses is surprising given the large volume of drugs handled and the potential health risks involved.

In this context, this paper reviews the design of a cluster, randomized controlled trial to evaluate the efficacy of an audit and feedback intervention to improve nurses’ use of personal protective equipment when handling hazardous drugs. The overall objective of this research program is to measure and improve the safety of chemotherapy administration in ambulatory oncology settings. The trial has three specific aims:

1. Evaluate the efficacy of an audit and feedback intervention to improve recommended use of PPE.
2. Determine whether the intervention effects on PPE use are mediated by knowledge about PPE use and perceived risk of hazardous drug exposure.
3. Determine whether the intervention effects on PPE use are moderated by personal (experience, education, and certification) and organizational factors (workloads, practice environments, and safety organizing behaviors).

Study results will inform practicing nurses, cancer center administrators, and policymakers on optimal approaches to protect workers who handle potentially hazardous drugs.

## Methods/Design

A cluster randomized controlled trial design was chosen to compare an educational module on hazardous drug handling with the same educational module plus feedback from survey and biological data obtained from participants. Specifically, a clustered design reduces the likelihood for contamination bias between participants and facilitates measurement of the organizational context that we hypothesize will moderate the effects of the proposed intervention. Participants will provide baseline data upon study enrollment. After evaluation for the primary endpoint is complete, all participants will receive the feedback materials for the remainder of the 4-year study. These materials and study questionnaires will be located on a user-authenticated website maintained by the investigative team. Table 1 shows the study procedures.

**Table 1:** **Drug Exposure Feedback and Education for Nurses' Safety (DEFENS) Study procedures**

	**Enrollment**	**Allocation**	**Post-allocation**							**Close-out**
TIMEPOINT (Month)	1-5	6	7	9	12	15	18	21	24	25-30
**ENROLLMENT**										
Eligibility screen	X									
Informed consent	X									
Site Coordinator Training	X									
Cluster Allocation		X								
**INTERVENTIONS**										
Control: Web-based educational module			X							
Intervention: Audit and Feedback			X					X (both arms)	X	X
Quarterly Reminders				X	X	X	X	X	X	X
**ASSESSMENTS**										
Baseline Survey Demographics, PPE use, Plasma levels, PPE Knowledge, PPE Barriers, Moderators		X								
Primary Endpoint PPE use, Plasma levels							X			
Spill Assessments PPE use, Plasma levels			X	X	X	X	X	X	X	X

PPE: Personal protective equipment.

### Human subjects considerations

The University of Michigan Institutional Review Board has approved the study (HUM00086541, date of last approval: 16 March 2015). All participants will complete informed consent on the study website. Of the 11 participating sites, 4 have reviewed study procedures and determined their staff to be 'not engaged' in the conduct of the research. Another three sites have ceded authority to the University of Michigan and the remaining four sites have pursued full protocol review. Recruitment will not begin at each site until local approvals have been obtained, as appropriate. A complete listing of human subjects approval is available (see Additional file 1).

### Conceptual framework

**Figure 1:**
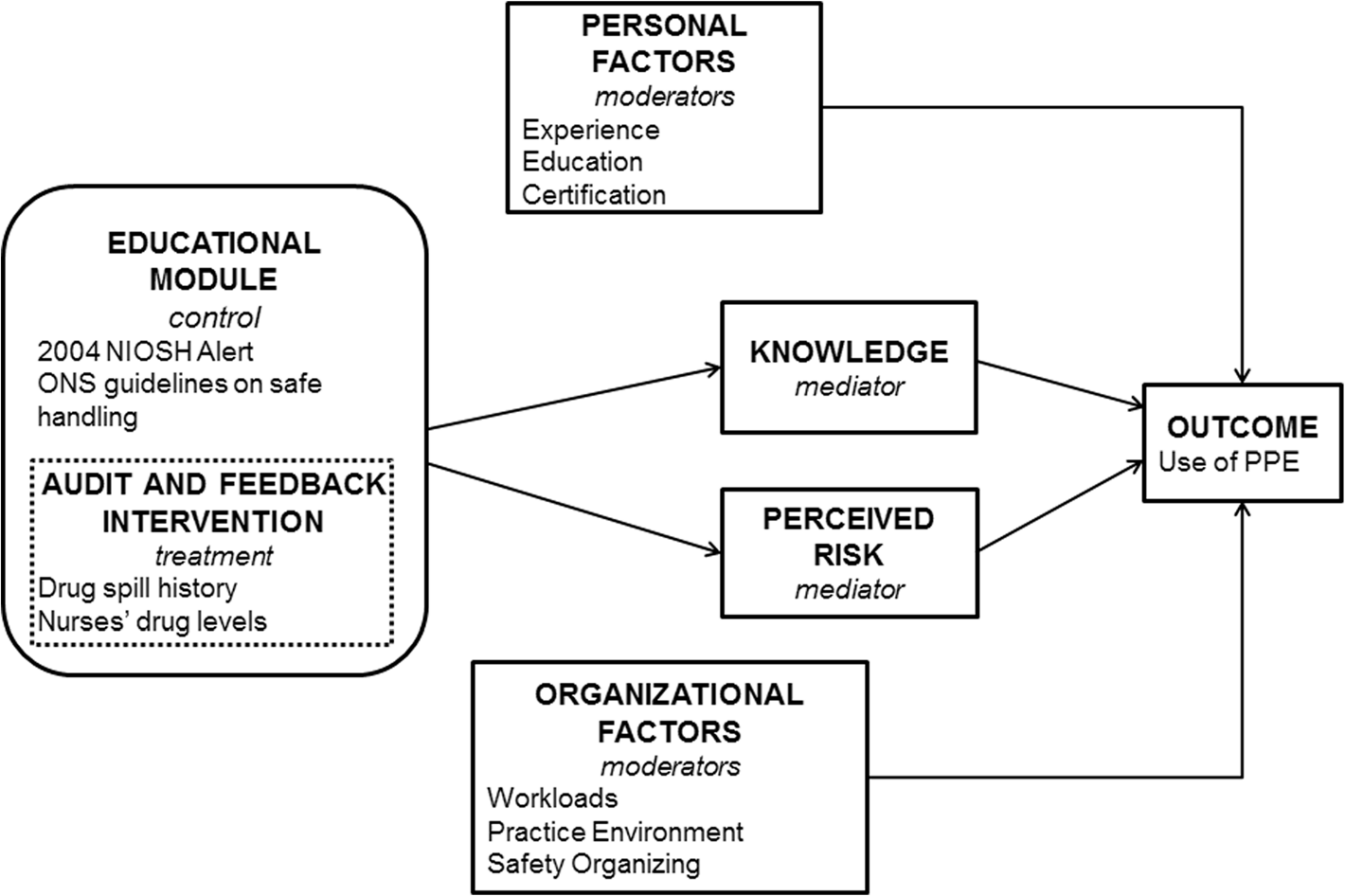
Conceptual framework. NIOSH: National Institute for Occupational Safety and Health. ONS: Oncology Nursing Society.

The study’s conceptual framework integrates theoretical, empirical, and pilot work that spans occupational health, health promotion, and organizational studies (Figure 1) [16-19]. The primary *outcome* of interest is PPE use using a valid and reliable measure described below [10]. The *interventions* are a 1-hour web-based educational module on hazardous drug safe handling with quarterly reminders about the educational content (control) or the web-based educational module with tailored messages plus quarterly feedback on hazardous drug spills and drug levels measured in the study population (treatment). Aim 1 will compare the control and treatment groups on PPE use. Next, we hypothesize the interventions will result in a) increased *knowledge* [20] about PPE use and b) increased *perceived risk* [21] of hazardous drug exposure. We hypothesize the treatment intervention will result in higher knowledge and perceived risk than the control intervention. In Aim 2, knowledge and perceived risk are considered mediators of the intervention effect because they are likely influenced by the intervention received and in turn will likely influence PPE use. Finally, preliminary work suggests a relationship between the *personal factor* of years of experience and PPE use. We will also explore additional personal factors, including nursing education and certification. Two *organizational factors* (nursing workloads and practice environments) are correlated to PPE use and hazardous drug exposure [11]. We will also explore a third factor of safety organizing behaviors [22]. In Aim 3, personal and organizational factors are considered moderators in our framework because they may strengthen or weaken the observed effect of the interventions on PPE use.

### Setting and sample

#### Settings

Site inclusion criteria are ambulatory oncology infusion settings with at least 20 employees who meet the eligibility criteria listed below. In addition, the chief nursing executive for cancer services in each facility provided endorsement of the study. Exclusion criteria are infusion areas that are not within easy access to the on-site study coordinator’s office or lack on-site phlebotomy services.

#### Sample

Primary inclusion criteria include registered nurses employed 16 hours or more per week in the ambulatory infusion area. To eliminate the chance of contaminated results in accordance with a previous protocol [23], exclusion criteria include treatment with an antineoplastic agent in the past year. Women who are pregnant will be allowed to participate but for safety reasons, will not have blood drawn.

#### Recruitment and retention

The principal investigator will visit each site and offer a live presentation that reviews study procedures. The presentation will be recorded so off-shift workers may view the material at their convenience. Our recruitment strategy incorporates procedures supported by Dillman’s Tailored Design Method [24]. Chief nursing executives for cancer services have endorsed the study and will co-sign all recruitment materials. Site leaders will provide a list of all nurses who meet employment criteria, and personalized Emails will be sent to each nurse by the site leader and co-signed by the principal investigator. An upfront $10 cash gift will be provided during the enrollment period. Each site will also have at least one study coordinator who is fully versed on the scientific protocol and who can direct study questions to the coordinating center.

To promote retention, we plan quarterly electronic updates to all sites through personalized Email messages from the coordinating center. The study website was developed by a professional vendor with expertise in user-centered design [25]. These efforts ensure study participants can navigate the website easily. At our post-intervention data collection point, participants will receive electronic and in-person cues to complete the survey, have blood draws performed, and receive a second $10 cash gift.

### Randomization

Randomization will occur after participants have enrolled and completed the baseline survey. Randomization will occur at the site, rather than the participant level, to reduce the likelihood of contamination across study arms within one cancer center. We recognize the sites vary by size. To address this, sites will be ordered by number of participants in decreasing order. The .ralloc command in Stata 12 (StataCorp, College Station, TX, USA) will perform random allocation in blocks of two so that one of the first two sites is in each condition. This will help ensure fairly equal sample size in the two groups.

### Education versus audit and feedback

Both the control and treatment interventions are delivered to individual nurse participants. Table 2 compares the control and treatment interventions.

**Table 2:** **Control and treatment interventions description**

	**Control**	**Treatment**
Format	Web-based	Web-based
Duration	Forty-five minutes of audio/video content	Sixty minutes of audio/video content
Content	Review of 2004 NIOSH alert and recommendations for practice	Content from control video + video messages from practicing nurses on strategies to reduce barriers to PPE use
Tailored Messaging	No	Yes: tailoring variables are barriers to PPE use measure obtained at baseline
Fidelity Assessment	Completion of post test	Completion of post test; paradata to track that video messages were viewed
Reminders	Email reminders every 3 months of content	Email messages every 3 months with updates on spill data collected

NIOSH: National Institute for Occupational Safety and Health; PPE: personal protective equipment.

#### Control: hazardous drug safe handling web-based educational module with quarterly reminders

Participants will view a 1-hour web-based educational module on safe handling procedures. Our study consultant will present a 1-hour informational webinar on principles of hazardous drug handling, consistent with Oncology Nursing Society chemotherapy guidelines [7] and recommendations from NIOSH [26] and the American Society of Health-System Pharmacists [8]. The module content includes a summary of the 2004 NIOSH alert regarding the health effects of hazardous drug handling, a summary of the recommendations for PPE use, and resources to identify whether a drug is classified as hazardous. Participants will complete a post test to measure knowledge of PPE and perceived risk of hazardous drug exposure. Continuing nursing education credit will be provided. Every 3 months, short messages that summarize one of the main points presented during the webinar will be viewable on the study website.

#### Treatment: tailored web-based educational module plus quarterly audit and feedback on spills and drug levels

For the tailored educational module, participants view the 1-hour module on safe handling procedures plus additional short videos tailored on the barriers to PPE use they reported in the baseline survey [27]. The videos address each barrier individually and offer suggestions for overcoming them. For example, a nurse who scores highly on the item, 'PPE makes me too hot' on the barriers questionnaire will view a video from an interviewed oncology nurse that has successfully addressed that barrier. The audit and feedback intervention is a video report prepared every 3 months during the study period. The report summarizes: (1) the number of drug spills reported, (2) the context of the spill occurrences (when, activities performed, pertinent details, and use of PPE), and (3) drug levels obtained from participants’ blood samples. The reports are viewable from the study’s secure website. Drug levels from our baseline assessment and from spills will be reported using procedures described below.

#### Intervention fidelity assessment

Fidelity of the control intervention will be assessed through the quiz required to receive a continuing education certificate. Fidelity of both treatments is expected to be high because delivery of content via a secure website assures consistent presentation. This mode of delivery offers greater fidelity than interventions delivered by a person, which can vary over time and with the person offering the intervention. Prior to accessing the site, participants will log on using their unique study identifier, allowing the study team to track the number of times each user accesses the site. Access (number of times) and duration (minutes viewed, longest time between keystrokes) data will be used in the analyses to assess intervention effects. Because randomization occurs at the site rather than the individual, there is minimal opportunity for crossover contamination bias. The use of unique, secure user logins and passwords limits access to the intended recipients only.

### Measures

Measures were selected for their fit to the conceptual framework of the study, their performance in previous studies, and documented validity and reliability (see Table 3).

**Table 3:** **Measures table and timing**

**Concept**	**Measure**	**Description**	**When collected**
Outcome	Revised Drug Handling Questionnaire	Five items, 0 to 5 (never to always) use selected PPE items	Baseline
			Primary endpoint
Mediator: Knowledge	Knowledge questionnaire	Twelve items, multiple choice, true/false about 2004 NIOSH alert and recommendations	Baseline
			After viewing module
			Primary endpoint
Mediator: Perceived Risk	Three items from Geer’s dermal exposure survey	Three items, score 1 to 4 (strongly disagree -strongly agree) about health risks from exposure	Baseline
			Primary endpoint
Personal Factor	Experience	Number of years in nursing, oncology nursing, and in current position	Baseline
Personal Factor	Highest education degree completed	Diploma, Associates’ Bachelors, Masters, or Doctorate	Baseline
Personal Factor	Completed certifications	ONS Chemotherapy certification, OCN, AOCN	Baseline
Organizational Factor	Workload	Number of patients cared for on shift	Baseline
			With a spill report
			Primary endpoint
Organizational Factor	Revised PES-NWI	Six subscales, 23 items, score range 0 to 5 (strongly disagree - strongly agree) about presence of favorable work features	Baseline
			Primary endpoint
Organizational Factor	Safety Organizing Scale	Nine items, score range 1 to 7 (not at all - to a very great extent) team performance of safety behaviors	Baseline
			Primary endpoint

AOCN: Advanced Oncology Certified Nurse; OCN: Oncology Certified Nurse; ONS: Oncology Nursing Society; PES-NWI: Practice Environment Scale of the Nursing Work Index.

Note. Baseline assessment begins in Year 1 of the study; the Primary endpoint is assessed approximately 18 months later.

#### Outcome

The study’s primary endpoint is optimal use of PPE, and it will be measured at the individual participant level using the Revised Hazardous Drug Handling Questionnaire originally developed by Martin and Larson [20] as modified by Polovich and Martin [10]. The items are mapped to the 2004 NIOSH alert recommendations [6]. Use of PPE is measured on a 6-point Likert scale (5 = always, 4 = 76 to 99% of the time, 3 = 51 to 75%, 2 = 26 to 50%, 1 = 1 to 25%, and 0 = never). A mean score is calculated for each participant across five items: use of chemotherapy gloves, double gloves, single-use disposable gowns, eye protection, and respirators. Higher scores reflect more frequent use of PPE elements. In the original study, test-retest Kappa was calculated at 0.80, and measure validity was established through direct observation of nurses who also completed the questionnaire. In a sample of 165 nurses who completed the revised scale, the Cronbach alpha was 0.83 [10]. Although the original scale asks separate questions about drug preparation, drug administration, and drug disposal activities, this study will focus on PPE use for drug administration only, an activity shared by all study participants. This measure will be obtained with the baseline survey and at the post-intervention assessment.

#### Potential mediators

Both knowledge of PPE and perceived risk of hazardous drug exposure are hypothesized to mediate the potential effects of the intervention on PPE use. These measures will be obtained at baseline at the individual participant level, after the educational module has been viewed, and at the post-intervention assessment. The mediator analysis will use the measures obtained after the intervention has been delivered. Both measures were validated by expert panel review and discussion with two focus groups of at-risk workers. In prior work, both measures achieved a content validity index of 1 from 3 experts [10].

Knowledge of PPE will be measured using a 10-item chemotherapy exposure questionnaire that assesses knowledge of the 2004 NIOSH alert. The measure was developed by a content expert and study consultant. Each item provides four answer choices with one correct answer for each question. The scale range is 0 to 10, with higher scores reflecting increased knowledge. Perceived risk of drug exposure will be measured using a 3-item subscale from Geer’s Occupational Dermal Survey [21]. A 4-point Likert scale (1 = *strongly disagree*, 4 = *strongly agree*) will be used to assess nurses’ perceptions of the risks of chemotherapy exposure and potential health effects. The score range is 1 to 4. The Cronbach alpha in a similar study population was 0.70 [10].

#### Potential moderators

Three organizational factors (workloads, practice environments, and safety organizing) and three personal factors (experience, education, and certification) are proposed moderators. These measures will be obtained on the web-based survey at baseline and the post-intervention assessment. In contrast to our outcomes and potential mediators, the moderators will be obtained from participants and aggregated to the cluster level.

Workloads will be measured by asking participants: 'How many patients did you assume primary responsibility for on your last shift?' For spill reporting, the time referent will be changed to 'the shift the drug spill occurred'. Workload measures correlate significantly with administratively-derived staffing levels and perceived staffing adequacy [28]. Workload is also significantly associated with patient mortality [29], nurse-reported needlesticks [30], and hazardous drug exposure [11].

Practice environments are workplace features that enable nurses to deliver high-quality care [31]. Items from the Practice Environment Scale of the Nursing Work Index, revised for ambulatory oncology, are scored on a 5-point Likert scale, where 1 = *strongly disagree* to 5 = *strongly agree* that the characteristic is present in the practice. The range of setting-level scores on a composite of the 6 subscales was 2.7 (disagree) to 5.0 (strongly agree). Previously analyzed for validity and reliability, acceptable fit was achieved in a structural equation model with a comparative fit index of 0.95 and a root mean-square error of approximation of 0.057, and subscale Cronbach alphas ranged from 0.80 to 0.90 [32]. Our preliminary data show lower scores (that is, poorer practice environments) for nurses who report hazardous drug spills. We will use the mean score of the 23-item composite measure for the proposed analyses (range = 1 to 5).

The Safety Organizing Scale [22] reflects behaviors employees perform in high-reliability organizations that avert operational failure. Nine items reflect the concept of a safety culture, and importantly, capture observable actions of clinicians. Each item is scored on a 7-point Likert scale (1 = *not at all*, 7 = *to a very great extent*) to reflect the degree to which the nurse and his/her co-workers engage in the behaviors on their work unit. The items identify safe performance as a function of five processes: preoccupation with failure, reluctance to simplify interpretations, sensitivity to operations, commitment to resilience, and deference to expertise. The scale has high internal reliability and discriminant validity [22].

Congruent with prior studies, the three potential moderators described above will be aggregated to the cluster level. In this study, a cluster is considered each of the 11 participating cancer centers. For each cluster, the mean value for these three measures will be calculated from the individual responses from each nurse in the cluster.

Three personal factors will be collected from each participant with the baseline survey: oncology nursing experience (years), education (diploma, associate’s degree, bachelor’s degree, master’s degree, post-master’s degree), and certification (Oncology Nursing Society chemotherapy certification, Oncology Certified Nurse, Advanced Oncology Certified Nurse, other certification).

### Baseline evaluation

After informed consent is obtained, participants will complete a baseline questionnaire online at the secure study website. Baseline blood draws will be performed on-site at the conclusion of a participant’s scheduled work shift. All plasma samples will be shipped to the University of Michigan for processing and analyses for the detection of 20 commonly-used chemotherapy drugs.

### Spill reporting

If a spill occurs in the ambulatory oncology infusion center throughout the 4-year study period, participants will return to the secure study website and complete a brief spill report. They will also have blood drawn at the end of the shift to obtain drug levels. A second blood draw will be performed 24 hours after the first one (or the next available business day) to obtain estimated peak and trough values, respectively.

### Plasma analyses

Participants will provide blood for plasma sampling at the baseline and post-intervention assessment, as well as with the occurrence of any reported drug spill during the study. The procedures below are used for all obtained samples. At the end of a nurse’s shift, the nurse will report to the participating site’s designated phlebotomy area. Trained and credentialed phlebotomy staff will perform venipuncture using standard technique and place whole blood into 5-mL heparinized tubes. Cells will be removed from plasma by centrifugation for 10 minutes at 1,000 to 2,000 × g using a refrigerated centrifuge. Plasma will then be pipetted into a clean polypropylene tube and stored in a −20°C or lower freezer. After plasma samples are frozen, they will be shipped by next-day air and on dry ice to the University of Michigan.

To measure levels of potentially hazardous drugs from the obtained samples, a specific and highly sensitive liquid chromatography-electrospray ionization-mass spectrometry (LC-ESI-MS) method will be established. We will focus our efforts on the 20 drugs that are the most commonly administered agents in ambulatory oncology settings with chemical properties suitable for analysis. However, as methods emerge for the measurement of other drugs, we will consider these as nurses report exposure outside of our original list of drugs. Signals from the test drug will be monitored under the multiple reaction monitoring mode of the LC-ESI-MS for quantification [33]. Ionization mode, precursor to product ion transition, ion source parameters (potential, gas, temperature, and so on), mobile phase, and column will be optimized and selected under direct infusion and flow injection analysis of the pure compound. The selection of extraction method, including protein precipitation, liquid-liquid extraction, and solid phase extraction, will depend on the drug properties (for example, acidity content, lipophilicity, aqueous solubility and chemical stability). The most efficient and specific extraction method will be used for sample preparation.

Drugs with similar properties and similar measurement methods (using the same column, same mobile phase, and similar extraction conditions) will be grouped into one method to simultaneously detect several drugs in one injection. This technique will greatly enhance the screening throughput. Each sample batch processed will include plasma samples from healthy, unexposed volunteers to ensure calibration. The established method for detecting multiple test drugs in one injection will be evaluated for linearity, specificity, and sensitivity according to guidance from the Food and Drug Administration [34]. Results from the LC-ESI-MS analyses will be entered into RedCAP, a secure, Health Insurance Portability and Accountability Act (HIPAA)-compliant cloud-based data management platform [35]. Specimens and survey data will be linked by unique study identifiers.

### Statistical analysis

Survey data will be stored on the password-protected, user-authenticated encrypted server behind a firewall. Our hypotheses are focused on the efficacy of an audit and feedback intervention to nurse participants. A total of 382 nurses are expected to be sampled from 11 sites. Each site is randomized into either control or intervention condition. Because the nurses within the same site are likely to show correlated outcomes, we will use linear mixed-effects models to account for the intraclass correlation for the proposed cluster randomized trial [36,37]. More specifically, we will use a random intercept model in which a variable *site* is created to identify the sites, and then adding *site* as a random effect to the mixed model.

Aim 1 evaluates the efficacy of audit and feedback to improve recommended PPE use (compared with an educational video). The outcome variable of the fixed-effect structure is the PPE use scores. The predictor of the fixed effect is the intervention indicator variable. The data from the PPE use questionnaire and demographics variables that are included in the fixed-effect structure to increase the precision of estimates. The random effect in the model is the *site* variable. We assume the *site* variable follows a normal distribution with mean zero and is independent of the error term in the mixed model. The hypothesis is that there will be a significant intervention effect such that nurses in sites receiving the treatment, in addition to the web-based educational module, will report higher PPE use scores compared to nurses in sites randomized to receive only the control. Means within time will be computed as descriptive statistics to help describe the effect.

Aim 2 will determine whether knowledge about PPE use and perceived risk of drug exposure mediate the effect of the treatment intervention on PPE use. The hypothesis is that the effect of the treatment on PPE use will be at least partially mediated by knowledge and perceived risk. To measure the mediation effect, we fit two linear mixed models. The first model is the same model we use in Aim 1. The second model adds the two potential mediators in the first model. The mediator effect is measured as the difference in the coefficients of the intervention variable between the two models. A 95% confidence interval is calculated for the estimate. If the confidence interval does not cross zero, it shows that mediation effect is statistically significant.

Aim 3 will determine whether the treatment intervention effect on PPE use is moderated (strengthened or weakened) by personal (experience, education, certification) and organizational factors (workloads, practice environments, safety organizing). We will use mixed model analyses that include receipt of the treatment intervention, the moderator variables, and the products of treatment intervention receipt with the moderator variables as predictors of PPE use. Significance tests of the product terms between moderator and intervention variables will indicate whether moderation is present [38]. We hypothesize that at least one of these moderator variables will interact significantly with the treatment intervention. When the product term is significant, we will conduct a *post hoc* analysis by plotting the PPE use versus intervention at various levels of the moderator variables. Using graphical presentations, we can show the size of the intervention effect and how the effects vary based on the values of moderate variables.

#### Sample size considerations and statistical power

The design and sample size for this study were determined in part by power analysis conducted by Optimal Design software [39] that is designed specifically for mixed models such as ours in which nurses are nested within sites that are treated as a random factor. We considered power for detecting a medium sized effect of the intervention and a medium sized multiple correlation (both as defined by Cohen) [40]. We considered scenarios with different numbers of clusters/sites, with different average numbers of nurses per site, and with different levels of the intraclass correlation coefficient (ICC) ranging from 0.01 to 0.03. The ICC will be a measure of the extent that PPE use differs across sites. The higher the ICC, the greater the sample size needed. ICCs of up to 0.03 are common; therefore, we aimed to obtain 80% power for tests with a 2-tailed alpha of 0.05 to detect medium sized effects with this ICC. Analysis revealed that we will obtain this power if our sample includes 11 sites with a mean of 26 participating nurses. In reality, our sites have a mean of 35 nurses (range of 20 to 90), suggesting that we will achieve 80% power even if we have a 25% decrease in the expected sample size (n = 287).

### Human subjects considerations

Potential participants will be invited to the study website using their unique assigned study identifier and will also complete informed consent. During the consent process, they will have a yes/no option of providing additional plasma and whole blood samples for our biorepository. A data safety monitoring board comprised of three faculty members not involved in the project will review study progress and human subjects concerns on a quarterly basis. Study withdrawals and potential adverse events will also be reviewed at this meeting and reported to our Institutional Review Board.

## Discussion

Despite three decades of data to suggest that nurses face health risks from suboptimal use of personal protective equipment when handling hazardous drugs, we have identified an alarming absence of tested interventions to improve practice. This cluster, randomized controlled trial will compare two interventions: an educational module with an educational module plus feedback that will improve nurses’ knowledge and reduce barriers to PPE use.

### Limitations

A key limitation to the project is the reliance on a self-report measure of PPE use as the primary endpoint. Resource constraints and the frequent application of PPE prohibit us from measuring PPE use through direct observation. However, the primary endpoint was validated in the original study with direct observation. A second limitation is our selection of elite cancer centers, as opposed to community-based oncology settings, will bias our results toward conservative exposure rates. The participating facilities are high-volume cancer centers that currently provide training and PPE to their staff. For an efficacy trial, larger samples of participants per cluster are needed. It is our goal to move from efficacy to effectiveness in a larger, more diverse sample of oncology practices that includes nurses and other health care workers who are at risk for drug exposure.

## Additional file

Additional file 1:
**Summary of human subjects approvals.** The file details the human subjects approvals process and outcome at all participating study sites.

## Abbreviations

DEFENS: Drug Exposure Feedback and Education for Nurses’ Safety
HIPAA: Health Insurance Portability and Accountability Act
ICC: Intraclass correlation coefficient
LC-ESI-MS: Liquid chromatography-electrospray ionization-mass spectrometry
NIOSH: National Institute for Occupational Safety and Health, Centers for Disease Control and Prevention, United States
PPE: Personal protective equipment
