# Supplementary material for: DEFENS - Drug Exposure Feedback and Education for Nurses’ Safety: study protocol for a randomized controlled trial
Source: Trials. 2015 Apr 17;16:171. doi: 10.1186/s13063-015-0674-5 (PMC4411718; doi:10.1186/s13063-015-0674-5)
Supplement: Additional file 1: — Summary of human subjects approvals. The file details the human subjects approvals process and outcome at all participating study sites. [file 13063_2015_674_MOESM1_ESM.docx]

**DEFENS – DRUG EXPOSURE FEEDBACK AND EDUCATION FOR NURSES SAFETY: STUDY PROTOCOL FOR A RANDOMIZED CONTROLLED TRIAL**

**SUMMARY OF HUMAN SUBJECTS APPROVALS**

The study protocol and all study activities were approved by the University of Michigan Institutional Review Board,

HUM00086541, latest approval date: March 16, 2015. The table below summarizes human subjects approvals at each participating site. Participant enrollment will not begin until appropriate local approvals are obtained.

| Institution | Status | Protocol Number |
| --- | --- | --- |
| Arthur G. James Cancer Hospital | Ceded to the University of Michigan | HUM00086541 |
| Fox Chase Cancer Center-Temple Health | Determined to not be engaged | N/A |
| Froedtert Hospital | Determined to not be engaged | N/A |
| H. Lee Moffitt Cancer Center & Research Institute | Under review | MCC 18148 |
| Karmanos Cancer Center | Under review | 014615B3E |
| Memorial Sloan-Kettering Cancer Center | Approved March 4, 2015 | 15-049 |
| Roswell Park Cancer Institute | Approved February 9, 2015 | I264914 |
| Smilow Cancer Hospital at Yale-New Haven | Determined to not be engaged | N/A |
| Stanford Healthcare | Determined to not be engaged | N/A |
| University of Kansas Cancer Center | Ceded to the University of Michigan | HUM00086541 |
| University of Kentucky Health Care | Ceded to the University of Michigan | HUM00086541 |
